# Performance of direct-from-blood-culture disk diffusion antibiotic susceptibility testing and its impact on antibiotic adjustment in bloodstream infections at a Malaysian tertiary center

**DOI:** 10.1128/spectrum.02863-24

**Published:** 2025-06-18

**Authors:** Chee Lan Lau, Ramliza Ramli, Petrick Periyasamy, Toh Leong Tan, Hui-min Neoh, Aliza Mohamad Yusof, Zainina Zainal Abidin, Noranati Zulkifli Chia, Mohd Syazwan Mohd Saaid, Munirah Abdul Aziz, Isa Naina-Mohamed

**Affiliations:** 1 Department of Pharmacology, Pharmacoepidemiology and Drug Safety Unit, Faculty of Medicine, Universiti Kebangsaan Malaysia 594610, Cheras, Kuala Lumpur, Malaysia; 2 Pharmacy Department, Hospital Canselor Tuanku Muhriz, Cheras, Kuala Lumpur, Malaysia; 3 Department of Medical Microbiology and Immunology, Faculty of Medicine, Universiti Kebangsaan Malaysia, Cheras, Kuala Lumpur, Malaysia; 4 Medical Department, Faculty of Medicine, Universiti Kebangsaan Malaysia, Cheras, Kuala Lumpur, Malaysia; 5 Department of Emergency Medicine, Faculty of Medicine, Universiti Kebangsaan Malaysia 594584, Cheras, Kuala Lumpur, Malaysia; 6 UKM Medical Molecular Biology Institute (UMBI), Universiti Kebangsaan Malaysia, Kuala Lumpur, Malaysia; 7 Faculty of Health Sciences, Universiti Kebangsaan Malaysia 69951 , Kuala Lumpur, Malaysia; 8 Department of Anaesthesiology & Intensive Care, Faculty of Medicine, Universiti Kebangsaan Malaysia 594577 , Cheras, Kuala Lumpur, Malaysia; City of Hope Department of Pathology, Duarte, California, USA

**Keywords:** disk diffusion, antibiotic susceptibility, bacteremia, positive blood culture, antimicrobial stewardship

## Abstract

Delayed antibiotics increase mortality in bloodstream infection (BSI). Direct-from-blood-culture disk diffusion antibiotic susceptibility testing (dAST) forecasts susceptibility earlier than conventional susceptibility testing (cAST). The study aimed to evaluate dAST performance and its impact on antibiotic adjustment in BSI. In this Malaysian single-center prospective study, dAST was performed and interpreted according to breakpoints by Clinical and Laboratory Standards Institute (CLSI) guidelines. The turnaround time (TAT), categorical agreements (CA), and predictive values of dAST for susceptibility by cAST were determined among positive blood cultures (PBC) between November 2022 and November 2023. The active and WHO AWaRe antibiotics administered before and after dAST results were compared. Of the 318 PBCs, the median of TAT was earlier than cAST by 35 hours. The CA for 3,561 organism-antibiotics combinations was 91.5%, with 3.6% very major errors (VME), 3.3% major errors (ME), and 5.2% minor errors (mE). The dAST achieved CA above 90% for *Staphylococcus aureus* with cefoxitin (98.5%), *Pseudomonas aeruginosa* with ceftazidime (100%), and *Acinetobacter baumannii* with ampicillin/sulbactam (100%). For Enterobacterales, most combinations were above 90%, including ampicillin (95.1%), ceftriaxone (95.7%), and meropenem (95.7%), though those of the aminopenicillin/inhibitor combinations were above 80%. Most errors were attributed to mEs. Among 159 BSI, the prescribing of active antibiotics improved significantly following dAST (73.0% versus 89.3%, *P* < 0.001) but not WHO Access antibiotics (35.8% versus 35.2%, *P* = 0.188). dAST had good CAs for most antibiotics, allowing earlier improvement in active antibiotics. The modest change in WHO Access antibiotics prescribing reveals practice gaps in need of antimicrobial stewardship.

**IMPORTANCE:**

Global deaths attributable to antimicrobial resistance are rising. Hence, rapid susceptibility testing is essential for timely antibiotic de-escalation to mitigate antimicrobial resistance (AMR) development from exposure to broad-spectrum antibiotics. Compared to the costly advanced technology, direct disk diffusion from blood culture (diffusion antibiotic susceptibility testing [dAST]) is an affordable method that can be quickly adopted. However, the reliability of dAST in informing susceptibility was mainly reported from Western countries and scarcely from other regions, including Southeast Asia, where the AMR burden is high. This study from Malaysia adds insights into the performance of dAST and the potential to apply it in similar resource-limited settings from the same region. Furthermore, assessing the dAST's influence on antibiotic prescribing identifies the gap in implementation to guide areas of improvement for optimizing clinical utility.

## INTRODUCTION

Bloodstream infection (BSI) is one of the leading health burdens associated with mortality and morbidity, and delays in active antibiotics further increase lethality (1). Recognizing the crucial demand for speed in organism identification and susceptibility to inform treatment, advancements in rapid diagnostics have since bloomed, shortening the time to results in hours and showing promising outcomes when coupled with antimicrobial stewardship (AMS) (2). However, such tools are often costly and inaccessible in resource-limited settings and those of low-middle-income countries (LMIC) where antibiotic resistance is high. Hence, the disk diffusion (DD) method for antibiotic susceptibility testing directly from positive blood culture (dAST), which can be set up easily with immediate implementation at low cost, offers an attractive alternative to costly molecular diagnostics to inform antibiotic susceptibility earlier (3). Several studies from Western countries demonstrated the reliability and clinical benefit of dAST in improving antibiotic timeliness and mortality (4–7), though some had conflicting findings (8). The unstandardized bacteria inoculum and manual operation rendered the variation in dAST performance (9). In addition, most reported categorical agreements (CA) and two (6, 10) evaluated the predictability values of dAST. However, LMIC and Southeast Asia regions are under-represented in the arena of rapid susceptibility testing, including the application of dAST. The current study aimed to evaluate the in-house dAST performance of antibiotic susceptibility results for blood pathogens from adult patients and to assess the antibiotic changes after dAST results in a Malaysian healthcare setting.

## MATERIALS AND METHODS

### Study design

**Fig 1:**
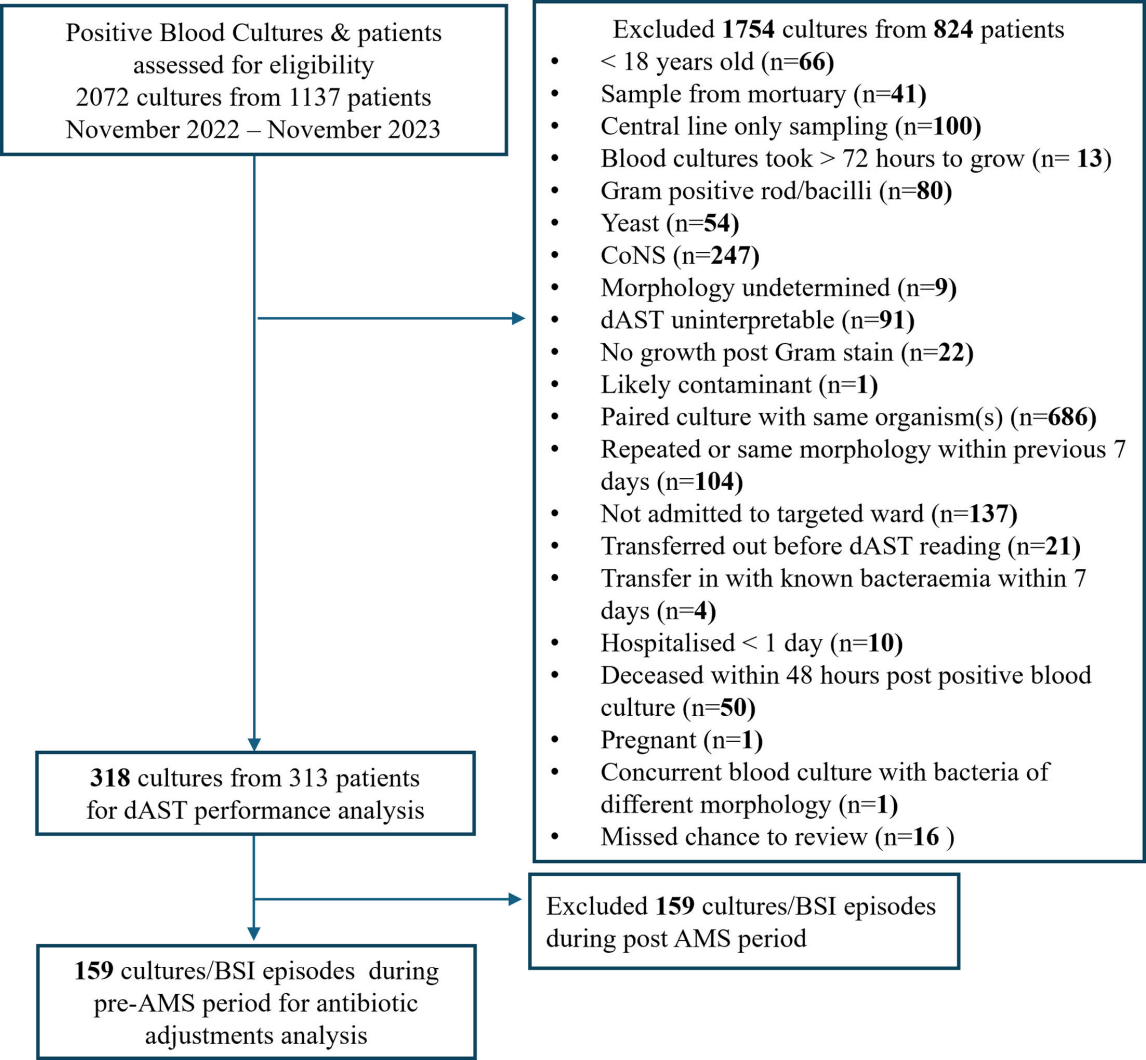
Enrolment of positive blood cultures.

This single-center prospective study was conducted at the Hospital Canselor Tuanku Muhriz, University Kebangsaan Malaysia (HCTM, UKM), a 1,054-bed tertiary care teaching hospital. Positive blood cultures (PBC) were screened between November 2022 and November 2023 for the following inclusion criteria: PBC from patients aged 18 years old and above who were admitted into wards under the care of medical, surgical, and intensive care specialties, availability of dAST readings and conventional susceptibility test (cAST) reports. Cultures from the same patient that were more than 7 days apart and morphologically different from the index organism were included. The first of the blood culture bottle pairs that turned positive was referred to in the dAST performance analysis. The cultures were excluded according to the criteria depicted in Fig. 1. The growth of common skin commensals such as coagulase-negative Staphylococci (CoNS), *Corynebacterium* spp., *Bacillus* spp. (other than *Bacillus anthracis*), *Micrococcus* spp., and *Cutibacterium acnes*/spp. was considered to be likely contaminants (11, 12). The AST was usually not performed by default unless requested by clinicians and agreed upon by the microbiologist. As repeated cultures were often required and the significance could not be determined when dAST results were read, the probable contaminants, including CoNS, were excluded from the dAST performance analysis.

### Susceptibility testing and reporting

Blood cultures from hospitalized patients were collected usually in a pair of BD BACTEC Plus Aerobic/F and BD BACTEC Lytic/10 Anaerobic/F culture vials and sent to the in-house HCTM’s diagnostic laboratory service as part of routine clinical care. The blood cultures received from 08:00 to 16:00 were loaded into a BD BACTEC FX system (Becton Dickinson, Franklin Lakes, NJ). The cultures sent after 16:00 were loaded the next morning. Gram staining and dAST were performed by the laboratory technicians for all positive blood cultures within 1 hour after a red flag signaling growth between 08:00 and 20:00 or the following morning for positive signals after 20:00. Gram stain results were verbally reported via phone to nurses or doctors.

dAST was performed for Gram-positive bacteria except for Gram-positive bacilli and all Gram-negative bacteria according to an in-house procedure using the unstandardized Kirby–Bauer disk diffusion method. Immediately after Gram staining, four drops of aliquots were taken from the PBC bottle to inoculate the Mueller Hinton blood agar and Mueller Hinton agar (MHA) (Isolab, Shah Alam, Malaysia). Selected antibiotic disks (Oxoid, Thermo Fisher Scientific, UK; or BD BBL Sensi-Disc, USA) were applied onto the MHA based on the Gram stain results with reference to the panels adapted from the Clinical and Laboratory Standards Institute documents (CLSI) M100 documents, 32nd and 33rd edition (13, 14): Gram-positive panel: penicillin (10 U), cefoxitin (30 µg), oxacillin (1 µg), clindamycin (2 µg), erythromycin (15 µg), gentamicin (10 µg), and ciprofloxacin (5 µg); trimethoprim-sulfamethoxazole (1.25/23.75 µg), doxycycline (30 µg), fusidic acid (10 µg), rifampicin (5 µg), mupirocin (200 µg), and linezolid (30 µg); Gram-negative panel: ampicillin (10 µg), gentamicin (10 µg), cefuroxime (30 µg), cefepime (30 µg), ciprofloxacin (5 µg), and ampicillin/sulbactam (10/10 µg); ceftazidime (30 µg), amoxicillin-clavulanate (20/10 µg), cefotaxime (30 µg), imipenem (10 µg), meropenem (10 µg), and ertapenem (10 µg), amikacin (30 µg), piperacillin/tazobactam (100/10 µg), and ceftriaxone (30 µg). The applied plates were incubated at 35°C ± 2°C ambient air for 16 to 18 hours. The zone diameter measurements and readings for dAST results were done once daily in the morning and were interpreted as susceptible (S), intermediate (I), and resistant (R) according to CLSI guidelines (13, 15). The dAST results and organisms’ identities were notified verbally by microbiologists via phone to nurses or doctors, and a note entry with the title “preliminary result” in the online Integrated Laboratory Management System (ILMS).

cAST results were used as a reference and were performed using two methods across the study period. The cAST, using the Kirby–Bauer DD method, was performed with the MHA inoculated after subculturing with a standard inoculum of 0.5 or 1.0 McFarland and incubated for 18 to 24 hours. The cAST was also done with the VITEK 2 Compact (bioMérieux, Marcy-l’Etoile, France) or VITEK 2 60 (bioMérieux, Marcy-l’Etoile, France) using AST-GP 67 or AST-N374 cards after subculturing with a standardized inoculum of 0.5 to 0.63 McFarland, according to the manufacturer’s instructions. The cAST results were interpreted by the microbiologists according to the latest CLSI M100 documents, 32nd and 33rd editions, as well as M45 documents 3rd edition (13–15). The finalized cAST reports with organism identification and susceptibilities were posted to the ILMS without verbal notification.

The organisms were identified by matrix-assisted laser desorption ionization-time of flight mass spectrometry (MALDI-TOF MS) (Bruker Daltonics, Bremen, Germany) or VITEK 2 system (bioMérieux, Marcy-l’Etoile, France), according to the manufacturer’s instructions. Mass spectrum analyses were referred to the database provided in MALDI BIOTYPER (Bruker Daltonics, Bremen, Germany), software version Compass 4.1.100, containing library version 12 and library number 11897. The definitive species identification of bacteria was based on the score value of ≥1.7.

### Turnaround time of dAST and cAST report

The turnaround time (TAT) was estimated from the time of the blood culture draw, flagged positive, to the time of reporting. The blood culture draw time was retrieved from the manual blood culture request forms. The recorded time of bottle removal from the incubator was referred to as the time of Gram staining. The dAST reporting time was retrieved from the manual entry in the bacteriology routine test worksheets or ILMS, whichever was earlier. If the time was missing, it was assumed to be at 10:00 on the date when the dAST was read, as the process of the dAST readings to reporting for the batch of the day is usually completed by 10:00 daily. The cAST reporting time followed the stated time in ILMS.

### Antibiotic modification after dAST results notifications

The impact of dAST results was determined by analyzing eligible PBCs from November 2022 to April 2023, when the AMS team was not involved. The patients’ medication charts were reviewed to observe the antibiotic changes for the antibiotics administered before and within 24 hours after dAST results notification, or before cAST results reporting time, and within 24 hours after cAST results. The antibiotics were categorized as active against the isolated pathogen based on the final susceptibility report by cAST (5, 8) and according to WHO AWaRe classification (16) as Access, Watch, and Reserve antibiotics.

### Statistical analysis

The CA and error rates between dAST and cAST results of susceptible (S), intermediate (I), or resistant (R), with reference to the standard breakpoints by CLSI guidelines, were calculated for each organism-antibiotic combination (Table 1) (17) . Organism-antibiotic combination(s) without dAST and/or cAST results were excluded. The categorical discrepancy rates of very major error (VME, susceptible by dAST and resistant result by cAST, < 3%), major error (ME, resistant by dAST and susceptible result by cAST, < 3%), and minor error (mE, disagreement between dAST and cAST for intermediate susceptible results from either test, ≤10%), were considered acceptable (18).

**TABLE 1:** Calculation of categorical agreement and error rates

Categorical agreement or errors	Formula	Term	Definition of term	Target^*a*^
Categorical agreement (CA)	(n_CA_ /N_total_) × 100	n_CA_	Total number of isolates with the matched results of “S” or “I” or “R” by dAST and cAST	≥90%
		N_total_	Total number of isolates tested with dAST and cAST results	
Minor error (mE)	(n_mE_ /N_total_) × 100	n_mE_	Total number of isolates with unmatched results for “I” by either dAST or cAST	≤10%
		N_total_	Total number of isolates tested with dAST and cAST results	
Major error (ME)	(n_ME_ /N_cAST.S_) × 100	n_ME_	Total number of isolates with the results of “R” by dAST and “S” by cAST	<3%
		N_cAST.S_	Number of isolates tested “S” by cAST	
Very major error (VME)	(n_VME_ /N_cAST.R_) × 100	n_VME_	Total number of isolates with the results of “S” by dAST and “R” by cAST	<3%
		N_cAST.R_	Number of isolates tested “R” by cAST	

^*a*^ Target is according to the CLSI document M52 (17) .

Descriptive data were described in frequency and percentage. Categorical data were analyzed using the Chi-squared or Fisher’s exact tests, where appropriate. The normality of continuous data was tested using the Shapiro-Wilk test. The analyses were done using Statistical Package for the Social Sciences, version 29.0 (IBM Corp, Armonk, NY, USA).

## RESULTS

A total of 318 PBCs from 313 patients were eligible for the dAST performance evaluation (Fig. 1). Of 1,754 cultures that were excluded, most were due to paired cultures and CoNS growth, followed by admissions from non-targeted wards and repeated cultures within 7 days.

### Categorical agreements and error rates of dAST

The overall CA of dAST with cAST for the total of 3,561 organism-antibiotic combinations was over 91.5% (3,259/3,561) with mE of 5.2% (186/3,561), ME of 3.4% (89/2,722) and VME of 3.6% (27/754) (Table 2) (cAST as DD for 2,096 combinations, CA 92.7%, mE 4.5%, ME 3.0%, and VME 2.2%, Table 3; VITEK-2 for 1,465 combinations, CA 89.8%, mE 6.3%, ME 3.7%, and VME 5.6%, Table 4). Most minor errors (71.5%, 133/186) arose from the dAST results showing intermediate against susceptible and resistant against intermediate by cAST.

**TABLE 2:** Overall categorical agreements and error rates for dAST^*a*^

Organism (*N*) and antibiotic	Number of isolates				Categorical agreement		Error rate					
	Total	S	I	R			VME		ME		mE	
					*n*	%	*n*	%	*n*	%	*n*	%
*Staphylococcus aureus* (65)												
Penicillin	65	12	0	53	65	100	0	0.0	0	0.0	0	0.0
Cefoxitin	65	51	0	14	64	98.5	1	7.1	0	0.0	0	0.0
Clindamycin	64	57	0	7	62	96.9	1	14.3	0	0.0	1	1.6
Sulfamethoxazole/trimethoprim	61	59	0	2	57	93.4	2	100	2	3.4	0	0.0
Doxycycline	64	57	0	7	59	92.2	1	14.3	3	5.3	1	1.6
Rifampicin	64	64	0	0	64	100	0	NA	0	0.0	0	0.0
Linezolid	62	62	0	0	62	100	0	NA	0	0.0	0	0.0
*Streptococcus* spp.^*b*^ (20)												
Penicillin	20	18	2	0	18	90.0	0	NA	0	0.0	2	10.0
Ampicillin	16	15	1	0	15	93.8	0	NA	0	0.0	1	6.3
Ceftriaxone	17	17	0	0	16	94.1	0	NA	1	5.9	0	0.0
Erythromycin	16	13	1	2	11	68.8	0	0.0	2	15.4	3	18.8
Gentamicin	1	1	0	0	1	100	0	NA	0	0.0	0	0.0
Vancomycin	2	2	0	0	2	100	0	NA	0	0.0	0	0.0
*Enterococcus* spp. (6)												
Penicillin	6	3	0	3	5	83.3	1	33.3	0	0.0	0	0.0
Ampicillin	5	4	0	1	4	80.0	0	0.0	0	0.0	1	20.0
Gentamicin	4	3	0	1	4	100	0	0.0	0	0.0	0	0.0
Linezolid	5	5	0	0	4	80.0	0	NA	1	20.0	0	0.0
Vancomycin	6	6	0	0	5	83.3	0	NA	0	0.0	1	16.7
Subtotal	543	449	4	90	518	95.4	6	6.7	9	2.0	10	1.8
Enterobacterales^c^ (187)												
Ampicillin	184	33	1	150	175	95.1	1	0.7	6	18.2	2	1.1
Amoxicillin/clavulanate	182	133	11	38	148	81.3	2	5.3	9	6.8	23	12.6
Ampicillin/sulbactam	182	117	11	54	151	83.0	3	5.6	8	6.8	20	11.0
Piperacillin/tazobactam	183	148	12	23	139	76.0	2	8.7	13	8.8	29	15.9
Cefepime	182	147	5	30	173	95.1	1	3.3	2	1.4	6	3.3
Ceftazidime	183	141	7	35	173	94.5	0	0.0	3	2.1	7	3.8
Cefotaxime	183	133	2	48	176	96.2	2	4.2	2	1.5	3	1.6
Ceftriaxone	184	140	0	44	176	95.7	2	4.5	4	2.9	2	1.1
Cefuroxime	184	116	4	64	167	90.8	1	1.6	6	5.2	10	5.4
Meropenem	184	175	0	9	176	95.7	0	0.0	2	1.1	6	3.3
Imipenem	184	169	2	13	168	91.3	2	15.4	2	1.2	12	6.5
Ertapenem	179	169	0	10	174	97.2	0	0.0	1	0.6	4	2.2
Ciprofloxacin	185	107	20	58	153	82.7	3	5.2	6	5.6	23	12.4
Amikacin	182	179	1	2	168	92.3	1	50.0	8	4.5	5	2.7
Gentamicin	183	162	3	18	168	91.8	0	0.0	5	3.1	10	5.5
Subtotal	2,744	2,069	79	596	2,485	90.6	20	3.4	77	3.7	162	5.9
*Klebsiella pneumoniae* (69)												
Amoxicillin/clavulanate	68	49	3	16	58	85.3	1	6.3	3	6.1	6	8.8
Ampicillin/sulbactam	68	45	3	20	59	86.8	0	0.0	4	8.9	5	7.4
Piperacillin/tazobactam	67	45	4	18	46	68.7	1	5.6	7	15.6	13	19.4
Cefepime	67	47	2	18	65	97.0	0	0.0	0	0.0	2	3.0
Ceftazidime	68	47	1	20	62	91.2	0	0.0	2	4.3	4	5.9
Cefotaxime	69	44	1	24	67	97.1	0	0.0	0	0.0	2	2.9
Ceftriaxone	67	47	0	20	63	94.0	0	0.0	3	6.4	1	1.5
Cefuroxime	69	41	1	27	63	91.3	0	0.0	2	4.9	4	5.8
Meropenem	69	61	0	8	63	91.3	0	0.0	1	1.6	5	7.2
Imipenem	69	61	0	8	65	94.2	0	0.0	2	3.3	2	2.9
Ertapenem	67	58	0	9	63	94.0	0	0.0	0	0.0	4	6.0
Ciprofloxacin	67	46	5	16	56	83.6	0	0.0	4	8.7	7	10.4
Amikacin	66	65	0	1	61	92.4	0	0.0	3	4.6	2	3.0
Gentamicin	68	62	0	6	64	94.1	0	0.0	1	1.6	3	4.4
Subtotal	949	718	20	211	855	90.1	2	0.9	32	4.5	60	6.3
*Escherichia coli* (92)												
Ampicillin	92	27	1	64	84	91.3	1	1.6	5	18.5	2	2.2
Amoxicillin/clavulanate	92	74	8	10	72	78.3	0	0.0	5	6.8	15	16.3
Ampicillin/sulbactam	92	64	6	22	72	78.3	3	13.6	3	4.7	14	15.2
Piperacillin/tazobactam	92	84	7	1	73	79.3	0	0.0	5	6.0	14	15.2
Cefepime	91	79	3	9	85	93.4	1	11.1	2	2.5	3	3.3
Ceftazidime	92	74	6	12	89	96.7	0	0.0	0	0.0	3	3.3
Cefotaxime	92	72	1	19	89	96.7	1	5.3	1	1.4	1	1.1
Ceftriaxone	92	73	0	19	91	98.9	0	0.0	0	0.0	1	1.1
Cefuroxime	92	67	3	22	82	89.1	0	0.0	4	6.0	6	6.5
Meropenem	92	92	0	0	91	98.9	0	NA	0	0.0	1	1.1
Imipenem	92	92	0	0	89	96.7	0	NA	0	0.0	3	3.3
Ertapenem	91	91	0	0	91	100	0	NA	0	0.0	0	0.0
Ciprofloxacin	92	48	12	32	74	80.4	3	9.4	2	4.2	13	14.1
Amikacin	92	91	1	0	86	93.5	0	NA	4	4.4	2	2.2
Gentamicin	91	82	0	9	85	93.4	0	0.0	3	3.7	3	3.3
Subtotal	1,377	1,110	48	219	1,253	91.0	9	4.1	34	3.1	81	5.9
*Pseudomonas aeruginosa* (21)												
Piperacillin/tazobactam	20	15	0	5	18	90.0	0	0.0	1	6.7	1	5.0
Cefepime	20	17	0	3	19	95.0	0	0.0	0	0.0	1	5.0
Ceftazidime	21	16	0	5	21	100	0	0.0	0	0.0	0	0.0
Meropenem	20	16	0	4	19	95.0	0	0.0	0	0.0	1	5.0
Imipenem	21	16	0	5	21	100	0	0.0	0	0.0	0	0.0
Ciprofloxacin	19	15	1	3	18	94.7	0	0.0	0	0.0	1	5.3
Amikacin	21	19	0	2	20	95.2	0	0.0	1	5.3	0	0.0
Gentamicin	19	17	0	2	19	100	0	0.0	0	0.0	0	0.0
Subtotal	161	131	1	29	155	96.3	0	0.0	2	1.5	4	2.5
*Acinetobacter baumannii*/ spp. (9)												
Piperacillin/tazobactam	9	4	0	5	8	88.9	0	0.0	0	0.0	1	11.1
Sulfamethoxazole/trimethoprim	7	3	0	4	7	100	0	0.0	0	0.0	0	0.0
Ampicillin/sulbactam	9	4	0	5	9	100	0	0.0	0	0.0	0	0.0
Meropenem	9	4	0	5	9	100	0	0.0	0	0.0	0	0.0
Imipenem	9	4	0	5	9	100	0	0.0	0	0.0	0	0.0
Amikacin	9	5	0	4	9	100	0	0.0	0	0.0	0	0.0
Gentamicin	9	5	0	4	8	88.9	0	0.0	1	20.0	0	0.0
Subtotal	61	29	0	32	59	96.7	0	0.0	1	3.4	1	1.6
*Burkholderia cepacia* (4)												
Gentamicin	1	0	0	1	1	100	0	0.0	0	NA	0	0.0
Ceftazidime	4	4	0	0	4	100	0	NA	0	0.0	0	0.0
Meropenem	4	4	0	0	2	50.0	0	NA	0	0.0	2	50.0
Amikacin	1	0	0	1	1	100	0	0	0	NA	0	0.0
Sulfamethoxazole/trimethoprim	4	4	0	0	4	100	0	NA	0	0	0	0.0
Subtotal	14	12	0	2	12	85.7	0	0.0	0	0.0	2	14.3
*Stenotrophomonas maltophilia* (2)												
Sulfamethoxazole/trimethoprim	2	2	0	0	2	100	0	NA	0	0.0	0	0.0
*Aeromonas hydrophila* (4)												
Gentamicin	4	2	1	1	2	50.0	0	0.0	0	0.0	2	50.0
Cefuroxime	2	2	0	0	2	100	0	NA	0	0.0	0	0.0
Cefepime	4	4	0	0	4	100	0	NA	0	0.0	0	0.0
Ciprofloxacin	4	4	0	0	2	50.0	0	NA	0	0.0	2	50.0
Ceftazidime	3	3	0	0	3	100	0	NA	0	0.0	0	0.0
Cefotaxime	3	3	0	0	3	100	0	NA	0	0.0	0	0.0
Imipenem	2	1	0	1	2	100	0	0.0	0	0.0	0	0.0
Meropenem	2	1	0	1	1	50.0	0	0.0	0	0.0	1	50.0
Ertapenem	1	1	0	0	1	100	0	NA	0	0.0	0	0.0
Amikacin	2	2	0	0	2	100	0	NA	0	0.0	0	0.0
Piperacillin/tazobactam	3	2	0	1	1	33.3	1	100	0	0.0	1	33.3
Ceftriaxone	3	3	0	0	3	100	0	NA	0	0.0	0	0.0
Sulfamethoxazole/trimethoprim	3	2	0	1	2	66.7	0	0.0	0	0.0	1	33.3
Subtotal	36	30	1	5	28	77.8	1	20.0	0	0.0	7	19.4
Grand total	3,561	2,722	85	754	3,259	91.5	27	3.6	89	3.3	186	5.2

^*a*^ S, susceptible; I, intermediate susceptible; R, resistant; VME, very major errors, susceptible by dAST and resistant by cAST; ME, major errors, resistant by dAST and susceptible by cAST; mE, minor errors, unmatched results of intermediate susceptibility by dAST or cAST. NA, not applicable.

^*b*^ Include *Streptococcus agalactiae* (*n* = 7), *Streptococcus anginosus* (*n* = 2), *Streptococcus dysgalactiae* (*n* = 4), *Streptococcus gallolyticus* (*n* = 3), *Streptococcus mitis* (*n* = 1), *Streptococcus parasanguinis* (*n* = 1), *Streptococcus sanguinis* (*n* = 1), and *Streptococcus pyogenes* (*n* = 1).

^*c*^ Include *Escherichia coli* (*n* = 92), *Klebsiella pneumoniae* (*n* = 69), *Proteus mirabilis* (*n* = 9), *Enterobacter cloacae* (*n* = 3), *Enterobacter hormaechei* (*n* = 1), *Klebsiella aerogene* (*n* = 2), *Klebsiella ozaenae* (*n* = 1), *Morganella morgannii* (*n* = 2)*, Providencia stuartii* (*n* = 1), *Serratia marcescens* (*n* =3), *Salmonella* spp. (*n* = 3), and *Plesiomonas shigelloides* (*n* = 1).

**TABLE 3:** Categorical agreements and error rates for dAST (reference method: disk diffusion)^*a*^

Organism (*N*) and antibiotic	Number of isolates				Categorical agreement		Error rate					
	Total	S	I	R			VME		ME		mE	
					*n*	%	*n*	%	*n*	%	*n*	%
*Staphylococcus aureus* (40)												
Penicillin	40	10	0	30	40	100	0	0.0	0	0.0	0	0.0
Cefoxitin	40	32	0	8	40	100	0	0.0	0	0.0	0	0.0
Clindamycin	39	34	0	5	38	97.4	1	20.0	0	0.0	0	0.0
Sulfamethoxazole/trimethoprim	37	37	0	0	35	94.6	0	NA	2	5.4	0	0.0
Doxycycline	40	35	0	5	38	95.0	1	20.0	1	2.9	0	0.0
Rifampicin	39	39	0	0	39	100	0	NA	0	0.0	0	0.0
Linezolid	38	38	0	0	38	100	0	NA	0	0.0	0	0.0
*Streptococcus* spp.^*b*^ (7)												
Penicillin	7	7	0	0	7	100	0	NA	0	0.0	0	0.0
Ampicillin	5	5	0	0	5	100	0	NA	0	0.0	0	0.0
Ceftriaxone	6	6	0	0	5	83.3	0	NA	1	16.7	0	0.0
Erythromycin	5	5	0	0	3	60.0	0	NA	1	20.0	1	20.0
Gentamicin	0	0	0	0	NA	NA	NA	NA	NA	NA	NA	NA
Vancomycin	1	1	0	0	1	100	0	NA	0	0.0	0.0	NA
*Enterococcus* spp. (4)												
Penicillin	4	1	0	3	3	75.0	1	33.3	0	0.0	0	0.0
Ampicillin	3	2	0	1	2	66.7	0	0.0	0	0.0	1	33.3
Gentamicin	4	3	0	1	4	100	0	0.0	0	0.0	0	0.0
Linezolid	4	4	0	0	3	75.0	0	NA	1	25.0	0	0.0
Vancomycin	4	4	0	0	3	75.0	0	NA	0	0.0	1	25.0
Subtotal	316	263	0	53	304	96.2	3	5.7	6	2.3	3	0.9
Enterobacterales^*c*^ (110)												
Ampicillin	109	16	0	93	104	95.4	0	0.0	4	25.0	1	0.9
Amoxicillin/clavulanate	107	78	7	22	86	80.4	1	4.5	6	7.7	14	13.1
Ampicillin/sulbactam	106	67	8	31	91	85.8	1	3.2	3	4.5	11	10.4
Piperacillin/tazobactam	107	84	11	12	81	75.7	1	8.3	7	8.3	18	16.8
Cefepime	105	83	3	19	101	96.2	1	5.3	1	1.2	2	1.9
Ceftazidime	106	81	3	22	103	97.2	0	0.0	1	1.2	2	1.9
Cefotaxime	106	76	1	29	102	96.2	2	6.9	1	1.3	1	0.9
Ceftriaxone	108	81	0	27	105	97.2	1	3.7	2	2.5	0	0.0
Cefuroxime	108	66	3	39	96	88.9	0	0.0	3	4.5	9	8.3
Meropenem	107	104	0	3	104	97.2	0	0.0	1	1.0	2	1.9
Imipenem	107	104	0	3	100	93.5	0	0.0	1	1.0	6	5.6
Ertapenem	103	100	0	3	102	99.0	0	0.0	0	0.0	1	1.0
Ciprofloxacin	108	68	7	33	93	86.1	0	0.0	4	5.9	11	10.2
Amikacin	107	105	1	1	100	93.5	0	0.0	4	3.8	3	2.8
Gentamicin	106	96	0	10	102	96.2	0	0.0	3	3.1	1	0.9
Subtotal	1,600	1,209	44	347	1,470	91.9	7	2.0	41	3.4	82	5.1
*Klebsiella pneumoniae* (42)												
Ampicillin	42	0	0	42	42	100	0	0.0	0	NA	0	0.0
Amoxicillin/clavulanate	41	33	2	6	35	85.4	0	0.0	2	6.1	4	9.8
Ampicillin/sulbactam	41	29	3	9	37	90.2	0	0.0	1	3.4	3	7.3
Piperacillin/tazobactam	41	29	3	9	29	70.7	1	11.1	4	13.8	7	17.1
Cefepime	40	30	1	9	40	100	0	0.0	0	0.0	0	0.0
Ceftazidime	41	31	1	9	38	92.7	0	0.0	1	3.2	2	4.9
Cefotaxime	42	29	0	13	42	100	0	0.0	0	0.0	0	0.0
Ceftriaxone	41	30	0	11	40	97.6	0	0.0	1	3.3	0	0.0
Cefuroxime	42	27	1	14	37	88.1	0	0.0	1	3.7	4	9.5
Meropenem	42	40	0	2	40	95.2	0	0.0	1	2.5	1	2.4
Imipenem	42	40	0	2	40	95.2	0	0.0	1	2.5	1	2.4
Ertapenem	40	38	0	2	39	97.5	0	0.0	0	0.0	1	2.5
Ciprofloxacin	40	32	1	7	35	87.5	0	0.0	2	6.3	3	7.5
Amikacin	41	40	0	1	39	95.1	0	0.0	1	2.5	1	2.4
Gentamicin	41	38	0	3	41	100	0	0.0	0	0.0	0	0.0
Subtotal	617	466	12	139	574	93.0	1	0.7	15	3.2	27	4.4
*Escherichia coli* (53)												
Ampicillin	53	14	0	39	48	90.6	0	0.0	4	28.6	1	1.9
Amoxicillin/clavulanate	53	40	5	8	41	77.4	0	0.0	3	7.5	9	17.0
Ampicillin/sulbactam	53	35	4	14	43	81.1	1	7.1	1	2.9	8	15.1
Piperacillin/tazobactam	53	46	7	0	40	75.5	0	NA	3	6.5	10	18.9
Cefepime	52	43	2	7	48	92.3	1	14.3	1	2.3	2	3.8
Ceftazidime	53	41	2	10	53	100	0	0.0	0	0.0	0	0.0
Cefotaxime	53	39	1	13	51	96.2	1	7.7	0	0.0	1	1.9
Ceftriaxone	53	40	0	13	53	100	0	0.0	0	0.0	0	0.0
Cefuroxime	53	35	2	16	46	86.8	0	0.0	2	5.7	5	9.4
Meropenem	53	53	0	0	52	98.1	0	NA	0	0.0	1	1.9
Imipenem	53	53	0	0	51	96.2	0	NA	0	0.0	2	3.8
Ertapenem	52	52	0	0	52	100	0	NA	0	0.0	0	0.0
Ciprofloxacin	53	28	4	21	45	84.9	0	0.0	2	7.1	6	11.3
Amikacin	53	52	1	0	49	92.5	0	NA	2	3.8	2	3.8
Gentamicin	52	47	0	5	49	94.2	0	0.0	2	4.3	1	1.9
Subtotal	792	618	28	146	721	91.0	3	2.1	20	3.2	48	6.1
*Pseudomonas aeruginosa* (13)												
Piperacillin/tazobactam	12	8	0	4	11	91.7	0	0.0	0	0.0	1	8.3
Cefepime	13	11	0	2	12	92.3	0	0.0	0	0.0	1	7.7
Ceftazidime	13	9	0	4	13	100	0	0.0	0	0.0	0	0.0
Meropenem	13	10	0	3	13	100	0	0.0	0	0.0	0	0.0
Imipenem	13	9	0	4	13	100	0	0.0	0	0.0	0	0.0
Ciprofloxacin	12	10	1	1	11	91.7	0	0.0	0	0.0	1	8.3
Amikacin	13	12	0	1	13	100	0	0.0	0	0.0	0	0.0
Gentamicin	12	11	0	1	12	100	0	0.0	0	0.0	0	0.0
Subtotal	101	80	1	20	98	97.0	0	0.0	0	0.0	3	3.0
*Acinetobacter baumannii/*spp. (7)												
Piperacillin/tazobactam	7	3	0	4	6	85.7	0	0.0	0	0.0	1	14.3
Sulfamethoxazole/trimethoprim	5	2	0	3	5	100	0	0.0	0	0.0	0	0.0
Ampicillin/sulbactam	7	3	0	4	7	100	0	0.0	0	0.0	0	0.0
Meropenem	7	3	0	4	7	100	0	0.0	0	0.0	0	0.0
Imipenem	7	3	0	4	7	100	0	0.0	0	0.0	0	0.0
Amikacin	7	4	0	3	7	100	0	0.0	0	0.0	0	0.0
Gentamicin	7	4	0	3	6	85.7	0	0.0	1	25.0	0	0.0
Subtotal	47	22	0	25	45	95.7	0	0.0	1	4.5	1	2.1
*Burkholderia cepacia* (4)												
Gentamicin	1	0	0	1	1	100	0	0	0	NA	0	0
Ceftazidime	4	4	0	0	4	100	0	NA	0	0	0	0
Meropenem	4	4	0	0	2	50.0	0	NA	0	0	2	50.0
Amikacin	1	0	0	1	1	100	0	0	0	NA	0	0
Sulfamethoxazole/trimethoprim	4	4	0	0	4	100	0	NA	0	0	0	0
Subtotal	14	12	0	2	12	85.7	0	0.0	0	0.0	2	14.3
*Stenotrophomonas maltophilia* (1)												
Sulfamethoxazole/trimethoprim	1	1	0	0	1	100	0	NA	0	0.0	0	0.0
*Aeromonas hydrophila* (2)												
Gentamicin	2	1	0	1	1	50	0	0.0	0	0.0	1	50.0
Cefuroxime	1	1	0	0	1	100	0	NA	0	0.0	0	0.0
Cefepime	2	2	0	0	2	100	0	NA	0	0.0	0	0.0
Ciprofloxacin	2	2	0	0	1	50.0	0	NA	0	0.0	1	50.0
Ceftazidime	1	1	0	0	1	100	0	NA	0	0.0	0	0.0
Cefotaxime	1	1	0	0	1	100	0	NA	0	0.0	0	0.0
Imipenem	1	1	0	0	1	100	0	NA	0	0.0	0	0.0
Meropenem	1	1	0	0	1	100	0	NA	0	0.0	0	0.0
Ertapenem	1	1	0	0	1	100	0	NA	0	0.0	0	0.0
Amikacin	1	1	0	0	1	100	0	NA	0	0.0	0	0.0
Piperacillin/tazobactam	1	1	0	0	0	0	0	NA	0	0.0	1	100
Ceftriaxone	2	2	0	0	2	100	0	NA	0	0.0	0	0.0
Sulfamethoxazole/trimethoprim	1	1	0	0	1	100	0	NA	0	0.0	0	0.0
Subtotal	17	16	0	1	14	82.4	0	0.0	0	0.0	3	17.6
Grand total	2,096	1,603	45	448	1,944	92.7	10	2.2	48	3.0	94	4.5

^*a*^ S, susceptible; I, intermediate susceptible; R, resistant; VME, very major errors, susceptible by dAST and resistant by cAST; ME, major errors, resistant by dAST and susceptible by cAST; mE, minor errors, unmatched results of intermediate susceptibility by dAST or cAST. NA, not applicable.

^*b*^ Include *Streptococcus agalactiae* (*n* = 3), *Streptococcus anginosus* (*n* = 1), *Streptococcus dysgalactiae* (*n* = 2), and *Streptococcus gallolyticus* (*n* =1).

^*c*^ Include *Escherichia coli* (*n* = 53), *Klebsiella pneumoniae* (*n* = 42), *Proteus mirabilis* (*n* = 3), *Enterobacter cloacae* (*n* = 3), *Klebsiella aerogene* (*n* = 1), *Klebsiella ozaenae* (*n* = 1), *Morganella morgannii* (*n* = 1), *Providencia stuartii* (*n* = 1), *Serratia marcescens* (*n* = 3), and *Salmonella* spp. (*n* = 2).

**TABLE 4:** Categorical agreements and error rates for dAST (reference method: VITEK-2, bioMérieux, Marcy-l’Etoile, France)^*a*^

Organism (*N*) and antibiotic	Number of isolates				Categorical agreement		Error rate					
	Total	S	I	R			VME		ME		mE	
					*n*	%	*n*	%	*n*	%	*n*	%
*Staphylococcus aureus* (25)												
Penicillin	25	2	0	23	25	100	0	0.0	0	0.0	0	0.0
Cefoxitin	25	19	0	6	24	96.0	1	16.7	0	0.0	0	0.0
Clindamycin	25	23	0	2	24	96.0	0	0.0	0	0.0	1	4.0
Sulfamethoxazole/trimethoprim	24	22	0	2	22	91.7	2	100	0	0.0	0	0.0
Doxycycline	24	22	0	2	21	87.5	0	0.0	2	9.1	1	4.2
Rifampicin	25	25	0	0	25	100	0	NA	0	0.0	0	0.0
Linezolid	24	24	0	0	24	100	0	NA	0	0.0	0	0.0
*Streptococcus* spp.^*b*^ (13)												
Penicillin	13	11	2	0	11	84.6	0	NA	0	0.0	2	15.4
Ampicillin	11	10	1	0	10	90.9	0	NA	0	0.0	1	9.1
Ceftriaxone	11	11	0	0	11	100	0	NA	0	0.0	0	0.0
Erythromycin	11	8	1	2	8	72.7	0	0.0	1	12.5	2	18.2
Gentamicin	1	1	0	0	1	100	0	NA	0	0.0	0	0.0
Vancomycin	1	1	0	0	1	100	0	NA	0	0.0	0	0.0
*Enterococcus* spp. (2)												
Penicillin	2	2	0	0	2	100	0	NA	0	0.0	0	0.0
Ampicillin	2	2	0	0	2	100	0	NA	0	0.0	0	0.0
Gentamicin	0	0	0	0	0	NA	0	NA	0	NA	0	NA
Linezolid	1	1	0	0	1	100	0	NA	0	0.0	0	0.0
Vancomycin	2	2	0	0	2	100	0	NA	0	0.0	0	
Subtotal	227	186	4	37	214	94.3	3	8.1	3	1.6	7	3.1
Enterobacterales^*c*^ (77)												
Ampicillin	75	17	1	57	71	94.7	1	1.8	2	11.8	1	1.3
Amoxicillin/clavulanate	75	55	4	16	62	82.7	1	6.3	3	5.5	9	12.0
Ampicillin/sulbactam	76	50	3	23	60	78.9	2	8.7	5	10.0	9	1.8
Piperacillin/tazobactam	76	64	1	11	58	76.3	1	9.1	6	9.4	11	14.5
Cefepime	77	64	2	11	72	93.5	0	0.0	1	1.6	4	5.2
Ceftazidime	77	60	4	13	70	90.9	0	0.0	2	3.3	5	6.5
Cefotaxime	77	57	1	19	74	96.1	0	0.0	1	1.8	2	2.6
Ceftriaxone	76	59	0	17	71	93.4	1	5.9	2	3.4	2	2.6
Cefuroxime	76	50	1	25	71	93.4	1	4.0	3	6.0	1	1.3
Meropenem	77	71	0	6	72	93.5	0	0.0	1	1.4	4	5.2
Imipenem	77	65	2	10	68	88.3	2	20.0	1	1.5	6	7.8
Ertapenem	76	69	0	7	72	94.7	0	0.0	1	1.4	3	3.9
Ciprofloxacin	77	39	13	25	60	77.9	3	12.0	2	5.1	12	15.6
Amikacin	75	74	0	1	68	90.7	1	100	4	5.4	2	2.7
Gentamicin	77	66	3	8	66	85.7	0	0.0	2	3.0	9	11.7
Subtotal	1,144	860	35	249	1,015	88.7	13	5.2	36	4.2	80	7.0
*Klebsiella pneumoniae* (27)												
Ampicillin	27	0	0	27	27	100	0	0.0	0	NA	0	0.0
Amoxicillin/clavulanate	27	16	1	10	23	85.2	1	10.0	1	6.3	2	7.4
Ampicillin/sulbactam	27	16	0	11	22	81.5	0	0.0	3	18.8	2	7.4
Piperacillin/tazobactam	26	16	1	9	17	65.4	0	0.0	3	18.8	6	23.1
Cefepime	27	17	1	9	25	92.6	0	0.0	0	0.0	2	7.4
Ceftazidime	27	16	0	11	24	88.9	0	0.0	1	6.3	2	7.4
Cefotaxime	27	15	1	11	25	92.6	0	0.0	0	0.0	2	7.4
Ceftriaxone	26	17	0	9	23	88.5	0	0.0	2	11.8	1	3.8
Cefuroxime	27	14	0	13	26	96.3	0	0.0	0	0.0	1	3.7
Meropenem	27	21	0	6	23	85.2	0	0.0	0	0.0	4	14.8
Imipenem	27	21	0	6	25	92.6	0	0.0	1	4.8	1	3.7
Ertapenem	27	20	0	7	24	88.9	0	0.0	0	0.0	3	11.1
Ciprofloxacin	27	14	4	9	21	77.8	0	0.0	2	14.3	4	14.8
Amikacin	25	25	0	0	22	88.0	0	NA	2	8.0	1	4.0
Gentamicin	27	24	0	3	23	85.2	0	0.0	1	4.2	3	11.1
Subtotal	401	252	8	141	350	87.3	1	0.7	16	6.3	34	8.5
*Escherichia coli* (39)												
Ampicillin	39	13	1	25	36	92.3	1	4.0	1	7.7	1	2.6
Amoxicillin/clavulanate	39	34	3	2	31	79.5	0	0.0	2	5.9	6	15.4
Ampicillin/sulbactam	39	29	2	8	29	74.4	2	25.0	2	6.9	6	15.4
Piperacillin/tazobactam	39	38	0	1	33	84.6	0	0.0	2	5.3	4	10.3
Cefepime	39	36	1	2	37	94.9	0	0.0	1	2.8	1	2.6
Ceftazidime	39	33	4	2	36	92.3	0	0.0	0	0.0	3	7.7
Cefotaxime	39	33	0	6	38	97.4	0	0.0	1	3.0	0	0.0
Ceftriaxone	39	33	0	6	38	97.4	0	0.0	0	0.0	1	2.6
Cefuroxime	39	32	1	6	36	92.3	0	0.0	2	6.3	1	2.6
Meropenem	39	39	0	0	39	100	0	NA	0	0.0	0	0.0
Imipenem	39	39	0	0	38	97.4	0	NA	0	0.0	1	2.6
Ertapenem	39	39	0	0	39	100	0	NA	0	0.0	0	0.0
Ciprofloxacin	39	20	8	11	29	74.4	3	27.3	0	0.0	7	17.9
Amikacin	39	39	0	0	37	94.9	0	NA	2	5.1	0	0.0
Gentamicin	39	35	0	4	36	92.3	0	0.0	1	2.9	2	5.1
Subtotal	585	492	20	73	532	90.9	6	8.2	14	2.8	33	5.6
*Pseudomonas aeruginosa* (8)												
Piperacillin/tazobactam	8	7	0	1	7	87.5	0	0.0	1	14.3	0	0.0
Cefepime	7	6	0	1	7	100	0	0.0	0	0.0	0	0.0
Ceftazidime	8	7	0	1	8	100	0	0.0	0	0.0	0	0.0
Meropenem	7	6	0	1	6	85.7	0	0.0	0	0.0	1	14.3
Imipenem	8	7	0	1	8	100	0	0.0	0	0.0	0	0.0
Ciprofloxacin	7	5	0	2	7	100	0	0.0	0	0.0	0	0.0
Amikacin	8	7	0	1	7	87.5	0	0.0	1	14.3	0	0.0
Gentamicin	7	6	0	1	7	100	0	0.0	0	0.0	0	0.0
Subtotal	60	51	0	9	57	95.0	0	0.0	2	3.9	1	1.7
*Acinetobacter baumannii/*spp. (2)												
Piperacillin/tazobactam	2	1	0	1	2	100	0	0.0	0	0.0	0	0.0
Sulfamethoxazole/trimethoprim	2	1	0	1	2	100	0	0.0	0	0.0	0	0.0
Ampicillin/sulbactam	2	1	0	1	2	100	0	0.0	0	0.0	0	0.0
Meropenem	2	1	0	1	2	100	0	0.0	0	0.0	0	0.0
Imipenem	2	1	0	1	2	100	0	0.0	0	0.0	0	0.0
Amikacin	2	1	0	1	2	100	0	0.0	0	0.0	0	0.0
Gentamicin	2	1	0	1	2	100	0	0.0	0	0.0	0	0.0
Subtotal	14	7	0	7	14	100	0	0.0	0	0.0	0	0.0
*Stenotrophomonas maltophilia* (1)												
Sulfamethoxazole/trimethoprim	1	1	0	0	1	100	0	NA	0	0.0	0	0.0
*Aeromonas hydrophila* (2)												
Gentamicin	2	1	1	0	1	50.0	0	NA	0	0.0	1	50.0
Cefuroxime	1	1	0	0	1	100.0	0	NA	0	0.0	0	0.0
Cefepime	2	2	0	0	2	100.0	0	NA	0	0.0	0	0.0
Ciprofloxacin	2	2	0	0	1	50.0	0	NA	0	0.0	1	50.0
Ceftazidime	2	2	0	0	2	100.0	0	NA	0	0.0	0	0.0
Cefotaxime	2	2	0	0	2	100.0	0	NA	0	0.0	0	0.0
Imipenem	1	0	0	1	1	100.0	0	0.0	0	NA	0	0.0
Meropenem	1	0	0	1	0	0.0	0	0.0	0	NA	1	100
Ertapenem	0	0	0	0	0	NA	0	NA	0	NA	0	NA
Amikacin	1	1	0	0	1	100.0	0	NA	0	0.0	0	0.0
Piperacillin/tazobactam	2	1	0	1	1	50.0	1	100.0	0	0.0	0	0.0
Ceftriaxone	1	1	0	0	1	100.0	0	NA	0	0.0	0	0.0
Sulfamethoxazole/trimethoprim	2	1	0	1	1	50.0	0	0.0	0	0.0	1	50.0
Subtotal	19	14	1	4	14	73.7	1	25.0	0	0.0	4	21.1
Grand total	1,465	1,119	40	306	1,315	89.8	17	5.6	41	3.7	92	6.3

^*a*^ S, susceptible; I, intermediate susceptible; R, resistant; VME, very major errors, susceptible by dAST and resistant by cAST; S, susceptible; I, intermediate susceptible; R, resistant; VME, very major errors, susceptible by dAST and resistant by cAST; ME, major errors, resistant by dAST and susceptible by cAST; mE, minor errors, unmatched results of intermediate susceptibility by dAST or cAST. NA, not applicable.

^*b*^ Include *Streptococcus agalactiae* (*n* = 4), *Streptococcus anginosus* (*n* = 1), *Streptococcus dysgalactiae* (n = 2), *Streptococcus gallolyticus* (*n* = 2), *Streptococcus mitis* (*n* = 1), *Streptococcus parasanguinis* (*n* = 1), *Streptococcus pyogenes* (*n* = 1), and *Streptococcus sanguinis* (*n* = 1).

^*c*^ Include *Escherichia coli* (*n* = 39), *Klebsiella pneumoniae* (*n* = 27), *Proteus mirabilis* (*n* = 6), *Enterobacter cloacae* (*n* = 1), *Klebsiella aerogene* (*n* = 1), *Morganella morgannii* (*n* = 1), *Salmonella* spp. (*n* = 1), and *Plesiomonas shigelloides* (*n* = 1).

Among Gram-positive organisms, the dAST results for *Staphylococcus aureus* (*S. aureus*) and *Streptococcus* spp. agreed with cAST well above 90% for all the combinations except for erythromycin (Table 2). One discrepant result for cefoxitin was due to borderline-oxacillin-resistant *S. aureus* (BORSA) when dAST and cAST by DD showed a zone diameter in the susceptible category, but the minimum inhibitory concentration was four by VITEK-2. The CA for ampicillin among *Enterococcus* spp. was lower, mainly due to one minor error for a susceptible *Enterococcus faecalis*.

Within the Enterobacterales, the CA ranged from 76.0% to 97.2%, mainly impeded by mE ranging from 1.1% to 15.9% (Table 2). Most antibiotics had CA above 90%, including ampicillin, third-generation cephalosporins (3GC), cefepime, and carbapenems. The CAs of aminopenicillin/β-lactam inhibitor antibiotics were above 80%, while those of piperacillin/tazobactam and ciprofloxacin were above 70%. The attributes of the errors were mostly mEs. The combinations with piperacillin/tazobactam had the lowest CA and mE rates. The combinations in *Klebsiella pneumoniae* (*K. pneumoniae*) and *Escherichia coli* (*E. coli*) shared a similar pattern of agreements (Table 2). Several antibiotic-Enterobacterales combinations had VMEs and MEs beyond the standard limits. Among the non-fermentative Gram-negative bacteria, the dAST achieved perfect agreements for ceftazidime and gentamicin in *Pseudomonas aeruginosa (P. aeruginosa*), ampicillin/sulbactam in *Acinetobacter* spp., as well as sulfamethoxazole/trimethoprim for *Burkholderia cepacia* and *Stenotrophomonas maltophilia* (Table 2).

### Turnaround time of dAST and cAST

**Fig 2:**
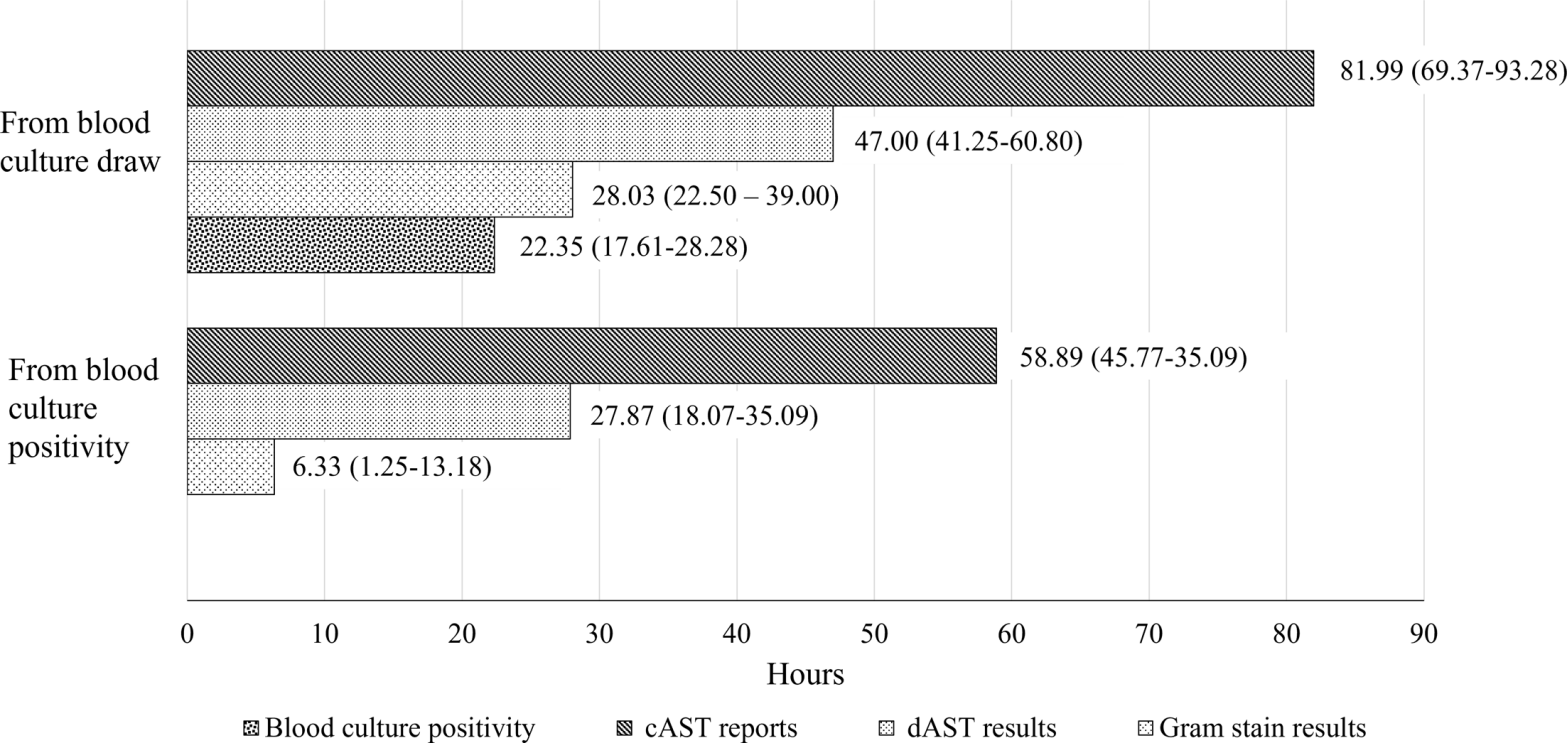
Turnaround time of dAST results and cAST reports (*N* = 318, time is expressed in median, interquartile range).

The median time from the blood culture draw to the dAST results was nearly 2 days and more than 24 hours earlier than the cAST report. When estimating from the time of flagged positivity, the median time from positivity was about a day to dAST results and more than 2 days to cAST report (Fig. 2).

### Antibiotics adjustment after dAST results and cAST reports

Of 159 BSIs reviewed, antibiotics were changed following dAST results in 96 (60.4%) episodes. Nearly three-quarters were given active antibiotics before, and the proportion improved significantly to well above 90% after the dAST results (Table 5). However, almost one-tenth remained on inactive antibiotics, including 13 BSIs for which dAST results had informed the likely susceptible antibiotics. Within 24 hours after cAST reports, the proportion of inactive antibiotics improved marginally. Watch antibiotics were administered in more than 60% of episodes, whereas Access antibiotics were persistently used in below half of BSIs without significant changes following dAST and cAST results.

**TABLE 5:** Antibiotic changes before, after dAST results, and after cAST reports (*N* = 159)^*a*^

	Before dAST, *n* (%)	After dAST, *n* (%)	*P* ^*f*^	After cAST, *n* (%)	*P^g^*
Receipt of *in vitro* active antibiotics					
Active antibiotics	116 (73.0)	142 (89.3)	<0.001^*e*^	146 (91.8)	0.566^*e*^
Inactive antibiotics	43 (27.0)	17 (10.7)^*b*^		13 (6.9)^*c*^	
AWaRe class prescribed					
Access antibiotics	57 (35.8)	56 (35.2)	0.188^*e*^	67 (41.1)	0.276^*e*^
Watch antibiotics	100 (62.9)	102 (64.2)		89 (56.0)	
Reserve antibiotics	0 (0)	1 (0.6)		1 (0.6)	
No antibiotics	2 (1.3)	0		2 (1.3)^*d*^	

^*a*^ Before dAST, between Gram stain result and dAST results time; after dAST, within 24 hours after dAST results and before cAST; after cAST reports, within 24 hours following cAST reports; Access antibiotics include ampicillin, amoxicillin/clavulanate, ampicillin/sulbactam, sulfamethoxazole-trimethoprim; Watch antibiotics include cefuroxime, ceftriaxone, cefepime, piperacillin/tazobactam, ertapenem, meropenem, imipenem, vancomycin; Reserve antibiotics include polymyxin B.

^*b*^ dAST results indicated resistant to existing antibiotics for all 17 cases.

^*c*^ Including four carbapenem-resistant isolates (*K. pneumoniae* and *Acinetobacter baumannii*) that were resistant to all the tested antibiotics in the panel and available options.

^*d*^ Antibiotics restarted beyond 24 hours after the cAST report.

^*e*^ Pearson Chi-square.

^*f*^ Between before and after dAST.

^*g*^ Between after dAST and after cAST, *P* < 0.05 indicates statistically significant.

## DISCUSSION

Compared to the previous studies that followed CLSI breakpoints, the CA rates are similar to the reported 92.3% to 96% in India (19) and China (10), in which the cAST was conventional DD. When the automated instrument was referred to as the cAST, the CA was closed to 87.6% in US children hospital (6) and in Canada (8), which employed automated broth microdilution method with Vitek-2 (bioMérieux) (6, 20), or MicroScan (Siemens) systems (8), respectively. The proportion of the total errors (8.5%, 302/3,561) in our study is consistent with the findings of 9.0% in Australia (20) but is higher than 2.1% in China (10). The variation in error rates was expected due to the different reference comparator methods and the blood culture incubators (9). Moreover, the difference in inoculation approach, such as using a sterile swab soaked with blood cultures (10) or drops, and the broth volume applied, ranging from one to four drops, could add uncertainty to the inconsistent inoculum density and cause the variation in the reported agreement rates (9). The inconsistent volume of two to four broth drops practiced by Daley P. et al. (8) might be one of the reasons for the overall unsatisfactory dAST agreements. Our study routinely applied four drops of aspirated broth as practiced by the CLSI group (9), Cao et al. (21), and Rajshekar et al. (19). Nevertheless, subjective appraisal by human readers with the naked eye could render differences in dAST performances across different laboratories.

Among Gram-positive bacteria, the agreement of the tested antibiotics panel in *S. aureus* was above the standard. The satisfactory agreement aligned with the previous findings (7, 19–21). One incidence of the cefoxitin dAST discrepancy was due to the resistant mechanism of BORSA, which is different from methicillin-resistant *Staphylococcus aureus* (MRSA), rendering the detection challenging (22). Regarding *Streptococcus* spp. and *Enterococcus* spp., the single very major error of penicillin in *Enterococcus* spp. was not an issue, as ampicillin was the preferred targeted antibiotic (23). Additionally, the collective error proportions of ampicillin (2/21, 9.5%) and penicillin (3/26, 11.5%) were mainly due to minor errors, as opposed to the proportions due to major errors in Menon et al. (20) and Rajshekar et al. (19).

Chandrasekaran et al. (9) highlighted that the dAST of β-lactams was prone to higher discrepancies due to the interaction with the blood components in the inoculum, hampering the antibiotics’ translocation to act on the bacteria. However, we observed satisfactory agreements for cefoxitin in *S. aureus*, cephalosporins, and carbapenems among the Gram-negative bacteria, consistent with Rajshekar et al. (19) and Wong et al. (10). Nevertheless, the just below standard agreement rates with the β-lactam/β-lactam inhibitors antibiotics in Enterobacterales accorded with the observations of 83.3% for piperacillin/tazobactam in Chandrasekaran et al. (9), 71.7% for ampicillin/sulbactam in Desai et al. (24), or 82.4% for amoxicillin/clavulanate in Edelmann et al. (25), respectively. However, it was higher than 52.8% in Daley et al. (8). Similarly, minor errors made up most of the disagreements.

Interestingly, the suboptimal CA of piperacillin/tazobactam in Enterobacterales and other Gram-negative bacteria in our study was consistent with previous studies, with the reported error fractions ranging from 7.6% to 17.4% (4, 8, 19, 20). Savage et al. (6) had an agreement above 95% for piperacillin/tazobactam after modifying the reference method from VITEK-2 to DD, but we found CA for piperacillin/tazobactam among Enterobacterales was above 70% with either VITEK-2 or DD as the reference method. Since the MERINO trial, the accuracy of the susceptibility testing for piperacillin/tazobactam was questioned, and the DD method was associated with higher error rates. Therefore, the cautions in interpreting cAST using the DD method (26) should be applied to the dAST results of piperacillin/tazobactam, acknowledging the potential high error rates.

Among the non-fermenters, previous investigations and ours universally reported a superior agreement for the relevant antibiotics against *P. aeruginosa* and *Acinetobacter* spp. (6, 19, 21). Although the included isolate numbers were small in our study, the perfect agreement of ceftazidime against *P. aeruginosa* or ampicillin/sulbactam and carbapenems against *Acinetobacter* spp. was in line with prior studies indicating the reliability of dAST to guide therapy in these two organisms.

Focusing on the WHO Access group antibiotics, our data showed that dAST had excellent agreements with low error rates for cefoxitin in *S. aureus*. This finding provides reassurance for therapy modification to cefazolin and potentially decreases the empirical vancomycin use for methicillin-susceptible *S. aureus*. Likewise, the dAST of ampicillin agreed well in *E. coli*, and that of amoxicillin/clavulanate exhibited reasonably good agreements, including *K. pneumoniae*. These are essential for decision-making upon the dAST notification of the susceptible likelihood to prompt early de-escalation and promote Access antibiotics use as advocated by the World Health Organization (16) before the cAST report.

Regarding the WHO Watch group antibiotics, the dAST predicted perfectly the ceftazidime susceptibility in *P. aeruginosa* in our study. Furthermore, the ceftriaxone and cefotaxime susceptibility also agreed well with the cAST of Enterobacterales isolates, including *E. coli* and *K. pneumoniae*, with reasonably low MEs. This suggests that dAST could reliably inform the likely absence of extended-spectrum β-lactamase (ESBL) and discourage empirical carbapenem continuation. However, the piperacillin/tazobactam non-susceptibility by dAST in Enterobacterales could do the opposite and require careful assessment, given the considerable false non-susceptibility results that yield high ME and mEs. The beyond-target VMEs of the 3GCs among the Enterobacterales were found mostly among the AmpC and ESBL-producing *Enterobacteriaceae*, which were noted for the likelihood during the dAST readings and prompted cefepime or carbapenem prescribing instead. Therefore, these did not negatively result in inactive antibiotics. The dAST of ertapenem and meropenem had good agreements with low error rates in anticipating carbapenem susceptibility in Enterobacterales isolates and *P. aeruginosa*. While the MEs and mEs might indicate the potential for the unnecessary alarm of carbapenem-resistant organisms, the dAST allows physicians to prepare for infectious disease consultation, seeking prior conditional approval to shorten the post-analytical time to antibiotics administration in genuine cases when the cAST report is available.

The current analysis of the CA and error rates was based on plain manual readings of the inhibition zones without incorporating the microbiologists’ interpretation. We did not analyze the agreements of ESBL and AmpC β-lactamase phenotypes, as this cannot be judged solely based on individual antibiotic disk and requires expert interpretation. CLSI M100 documents (13, 14) recommend reporting susceptible or intermediate readings as resistant for derepressed AmpC, ESBL, or carbapenemase-producing organisms. Incorporating these rules into the analysis could significantly reduce errors and improve the agreements and accuracy of beta-lactam dAST results (27). The large proportion of mE due to the discrepant dAST results with lower susceptibility categories than that of cAST aligns with the findings by Chandrasekaran et al. (9). This might be attributed to the smaller inhibition zone diameters after shorter incubation durations, likely for those with dAST processed at the later part of the previous day. Therefore, this could result in dAST readings of intermediate susceptibles disagreeing with cAST for susceptible isolates, referring to the CLSI breakpoints for incubated bacterial colonies at standard McFarland over a standard duration. Adjusting the inhibition diameter breakpoints according to the incubation period could reduce the error rates, as proposed by Cao et al. (21).

The TAT for the dAST test was as expected for a diagnostic test directly from blood culture, bypassing subculturing to allow results to be reported at least 24 hours earlier than the conventional method (9). The median duration from blood culture draw to dAST reporting in our study was similar to the median 41 hours (interquartile range, IQR 36–47 hours) in Jhaveri et al. (7) but longer than the reported 26.7 hours (IQR 22.5–28.6 hours) in Reiber et al. (28) likely because the latter was using an automated machine to perform readings after 6 hours incubation. Moreover, our laboratory workflow was non-round-the-clock, with once-daily plate readings. However, the lapse in loading the blood culture bottles sent after office hours into the incubator might prolong the time to positivity (29). Hence, the interval could be shorter with frequent readings and 24 hour laboratory service (30). Nevertheless, the faster TAT might not always translate into faster antibiotic adjustments, as Reiber et al. (28) noted, in which therapy was only de-escalated 28.7 hours after reporting, or Bhalodi et al. (31) with 24/7 hour laboratory operation in which the time to optimal therapy took 23.7 hours when the TAT was only 7 hours. Multimodal approaches and multidisciplinary collaboration are required to exert the intended purpose of rapid results (32).

Our current data only reports the antibiotic adjustments as the effect of the dAST results with the existing notification mechanism from November 2022 to April 2023, when the AMS collaboration was not yet integrated. Similar to other studies in Western countries (6, 8, 20, 28, 33), less than half of the antibiotics were modified following dAST results, likely because most were given active antibiotics prior. However, not all antibiotics were switched immediately to the active option following dAST notification and even cAST reports. The same was observed in the Turkish study (5) for 10% (5/49) of the BSI caused by ESBL or carbapenemase-producing Enterobacterales. One possible explanation could be the prescribing attitude, which the prescribers might not have the urgency to switch when patients were clinically stable (34). Nevertheless, this is a cause of concern as each day of delay in active antibiotics was associated with an incremental risk of death (1, 35) and prolonged hospital stay (36). Additionally, the proportion of Access antibiotics only reached half of the benchmark set by the World Health Organization (16), which was 60% of the overall antibiotics use. The exposure to Watch antibiotics had higher odds than Access antibiotics to precipitate infection or colonization by multidrug-resistant organisms (37). Further analysis is needed to determine the antibiotics’ appropriateness and the opportunities to improve Access antibiotics use. Ultimately, the observed patterns of antibiotic adjustment reveal practice gaps in need of AMS (2).

The limitations of our study are similar to those of Savage et al. (6) in that the low frequency of isolates restrained the agreement precision of various organisms, such as *P. aeruginosa* and *Streptococcus* spp. The determination of VME might be better in a setting with a higher resistance rate. Furthermore, the dAST readings were retrieved from manually documented categorical results without specifying zone diameters, for which the possibility of transcribing errors was uncertain. Besides, the study was conducted in a single-center academic tertiary care setting, which might not be generalizable to other sites with different infrastructures and expertise.

### Conclusion

The current study adds to the increasing data that dAST results have good agreement with cAST for antibiotic susceptibility at least 1 day before cAST reports, allowing earlier improvement in active antibiotic use. However, further measures such as AMS integration are needed to act upon the availability of dAST results for consistent and appropriate antibiotic prescribing earlier. These findings from LMIC highlight the potential of dAST to be adopted in similar resource-limited settings.

## Data Availability

The original contributions presented in the study are included in the article. Further inquiries can be directed to the corresponding author.
